# Morusin as a drug candidate: opportunities, limitations, and the path toward clinical translation

**DOI:** 10.3389/fphar.2025.1704535

**Published:** 2025-11-14

**Authors:** Hany N. Azzam, Ahmed M. El-Dessouki, Kareem A. Attallah, Mohamed A. Sadek, Yara M. Aboulmagd, Mennat-Allah M. Hassan, Mohamed I. Fahmy, Riham A. El-Shiekh, Rania M. Kamal, Hazim O. Khalifa

**Affiliations:** 1 Pharmacy Practice Department, Faculty of Pharmacy, Heliopolis University, Cairo, Egypt; 2 Pharmacology and Toxicology Department, Faculty of Pharmacy, Ahram Canadian University, Giza, Egypt; 3 Research and Development Department, Biotechnology Research Center, New Damietta, Egypt; 4 Clinical Research Department, Damietta Directorate for Health Affairs, Egyptian Ministry of Health and Population, Damietta, Egypt; 5 Department of Pharmacology and Toxicology, Faculty of Pharmacy, Cairo University, Cairo, Egypt; 6 Department of Pharmacology and Toxicology, College of Medicine, University of Arkansas for Medical Sciences, Little Rock, AR, United States; 7 Department of Pharmacology & Toxicology Department, Faculty of Pharmacy, Heliopolis University, Cairo, Egypt; 8 Department of Pharmacology and Toxicology, College of Pharmaceutical Sciences and Drug Manufacturing, Misr University for Science and Technology (MUST), Giza, Egypt; 9 Department of Pharmacognosy, Faculty of Pharmacy, Cairo University, Cairo, Egypt; 10 Department of Veterinary Medicine, College of Agriculture and Veterinary Medicine, United Arab Emirates University, Al Ain, United Arab Emirates

**Keywords:** morusin, Morus alba, prenylated flavone, nutraceuticals, pharmafood, health effects, anticancer, antimicrobials

## Abstract

Prenylflavonoids are widespread in plants, which are considered valuable sources of natural polyphenolic compounds with isoprenyl groups, including flavones, flavanones, chalcones and aurones. Among the notable prenylated flavonoids, morusin-a prenylated flavone isolated from the bark of white mulberry, has garnered attention for its multifaceted biological activities. Extensive research has demonstrated that morusin exhibits pronounced analgesic, antioxidant, anti-inflammatory, bone repair, antitumor, cardioprotective, neuroprotective, hepatoprotective, antidiabetic, and antimicrobial effects. The enhanced lipophilicity imparted by prenylation is believed to facilitate greater cellular membrane interaction, contributing to the superior bioactivity of these compounds compared to their non-prenylated derivatives. Unlike previous reviews that mainly emphasize morusin’s bioactivities, this article critically addresses its pharmacokinetic limitations, translational challenges, and safety concerns, offering a more integrated perspective on its path toward clinical application. This review aims to get current insights into the health-promoting effects of morusin, thereby informing the development of novel plant-derived pharmaceuticals and nutraceuticals within the prenylflavonoid category.

## 1 Introduction

Interest in natural products as sources for drug leads is experiencing a significant resurgence in contemporary pharmaceutical research (Abd El-Hafeez et al., 2018; Jahan Oni et al., 2024; Bithi et al., 2025; Eity et al., 2025; Rakib et al., 2025). Several articles have emphasized that natural products and their isolated compounds continue to play a vastly significant part in the drug discovery and development process (Attallah et al., 2023; Aboutaleb et al., 2025; El-Shiekh et al., 2025; Elbadry et al., 2025; Mustafa et al., 2025). Morusin is a naturally occurring prenylated flavone predominantly isolated from the root cortex of *Morus alba* L. (Moraceae). Structurally, it is characterized by a flavone backbone bearing hydroxyl groups at positions C-5, C-2′, and C-4′, a prenyl group at C-3, and a 2,2-dimethylpyran ring spanning C-7 and C-8 (Agarwal et al., 2019). These structural features, particularly the prenylation and pyran ring substitution, enhance its lipophilicity and are thought to contribute significantly to its pharmacological potency and interaction with biological membranes (Morante-Carriel et al., 2024).

Pharmacologically, morusin has attracted considerable attention for its broad spectrum of bioactivities. It has been reported to exert anticancer, antioxidant, anti-inflammatory, hepatoprotective, neuroprotective, cardioprotective, antimicrobial, and even antiviral effects (Jia et al., 2020; Martins et al., 2021; Panek-Krzyśko and Stompor-Gorący, 2021; Zhou et al., 2021). In oncology research, morusin demonstrates cytotoxic and growth-inhibitory effects across multiple cancer cell lines, including breast, liver, lung, prostate, colorectal, and gastric cancers, often through the modulation of apoptosis, autophagy, and key signaling pathways such as NF-κB, STAT3, and PI3K/AKT (Panek-Krzyśko and Stompor-Gorący, 2021). Beyond oncology, its antioxidant and anti-inflammatory actions support potential applications in chronic inflammatory diseases, neurodegeneration, cardiovascular dysfunction, and infectious disease management. Collectively, these multifaceted activities underscore morusin’s promise as a lead compound in drug discovery.

Despite this growing pharmacological evidence, the translation of morusin from bench to bedside remains limited. Most existing reviews emphasize its therapeutic potential while overlooking the crucial barriers that hinder clinical application. These include poor aqueous solubility, limited bioavailability, scarce pharmacokinetic and toxicological data, and the complete absence of clinical trials. Moreover, mechanistic gaps remain—for instance, while its antibacterial mechanisms have been partly elucidated, its antifungal actions are poorly understood, leaving important questions unanswered regarding resistance development and target specificity.

What sets this review apart is its critical focus on these overlooked but essential aspects, namely, the compound’s poor bioavailability, the absence of comprehensive human data, and the pressing need for multidisciplinary strategies to overcome these barriers. By integrating current pharmacological evidence with translational challenges, this review aims to provide a more holistic and realistic perspective on morusin’s clinical prospects, paving the way for future innovations that could turn its therapeutic promise into practical medical applications.

## 2 Search strategy

The data in the available literature has been collected from the databases including the Egyptian Knowledge Bank database, ScienceDirect, Scopus, Web of Science, PubMed, Springer, Google Scholar, Emerald, and Elsevier databases. The articles from Database journals were limited to research papers with the keywords “morusin” and “natural origins”, “isolation” and “natural sources or, solubility or, synthesis” or “mechanism of actions/biological activities and applications” or “clinical surveys” or “pharmacokinetics and bioavailability”. There was no defined time frame for data collection; all relevant data were gathered comprehensively. Articles are selected based on the following criteria: quality criteria during selection of studies and scoping of research, illustrative research article, high reproducibility, inventive potential within articles in morusin and its applications, articles in morusin and its biological activities, and articles with pharmacological evidence.

## 3 Natural origin, chemical characteristics, and analytical profile of morusin

### 3.1 Natural sources of morusin

Morusin is one of the prenylated flavonoids, specifically belonging to prenylated flavones. Prenylated flavonoids are polyphenolic compounds that contain isoprenyl groups attached to their basic flavonoid skeleton. These compounds are found in nature, such as in various plant parts, including flowers, bark, and stems (Nomura et al., 1983; Panek-Krzyśko and Stompor-Gorący, 2021). These compounds undergo the prenylation process, which involves the addition of isoprene units (C_5_H_8_) to the flavonoid skeleton. This process significantly alters the biological activities of these compounds. Morusin is predominantly found in various species of the *Morus* genus (mulberry) with significant concentrations in the roots of a leafy tree native to Southeastern Asia, (*M. alba* [White Mulberry]) (Panek-Krzyśko and Stompor-Gorący, 2021). Morusin was also reported to be found in a considerable amount in (*Morus nigra* [Black Mulberry]) roots (Zoofishan et al., 2018; Ferraz et al., 2024).

### 3.2 Chemical structure

**FIGURE 1:**
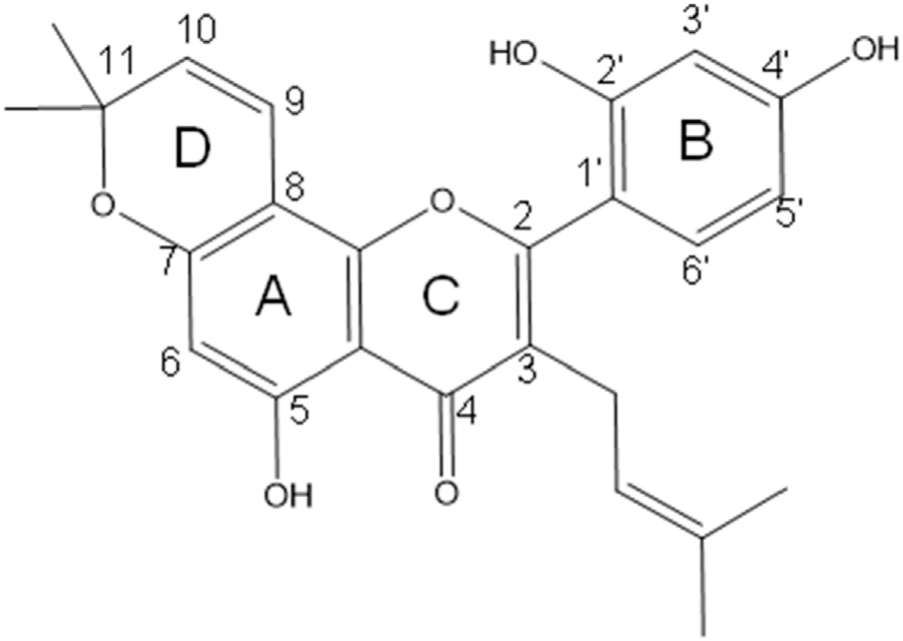
Chemical structure of morusin, featuring a flavone core with three aromatic rings (A–C), hydroxyl groups at C5, C2′, and C4′ positions, and two prenyl units including an isoprenoid substituent on ring C and a dimethylpyran ring formed between C7 and C8, contributing to its unique bioactivity.

Morusin chemical structure consists of the known flavone skeleton with additional prenyl groups. its molecular structure consists of three aromatic rings (A–C), featuring a hydroxyl group at the C5 position of ring A, and two hydroxyl groups at the C2′ and C4′ positions of ring B. Additionally, it contains two prenyl units: one isoprenoid substituent attached at C3 of the oxygenated ring C, and another forming a dimethylpyran ring (ring D) by bridging positions C7 and C8 (Figure 1). Its IUPAC name is 2-(2,4-dihydroxyphenyl)-5-hydroxy-8,8-dimethyl-3-(3-methylbut-2-en-1-yl)-4H,8H-pyrano [2,3-f]chromen-4-one (Chan et al., 2019; Choi et al., 2020).

### 3.3 Isolation and spectroscopic characterization

Plant material containing morusin is typically subjected to ethanol or methanol extraction, followed by filtration and concentration under reduced pressure. To isolate morusin from other flavonoids, further purification is commonly achieved using column chromatography (e.g., silica gel, Sephadex LH-20) or preparative high-performance liquid chromatography (HPLC) (De Souza et al., 2000; Tan et al., 2025; Tan et al., 2025). While recent advancements such as enzyme-assisted extraction and magnetic nanoparticle-based techniques have been reported for other prenylflavonoids (Li et al., 2018), these approaches have not yet been applied specifically to morusin.

Several spectral data for morusin have been documented. Ultraviolet–visible (UV–Vis) spectroscopy shows absorption maxima characteristic of flavones in the range of 250–350 nm. Fourier-transform infrared spectroscopy (FTIR) reveals peaks for hydroxyl (–OH), carbonyl (C=O), and prenyl (C–H stretching in isoprenoid groups) functional groups. In nuclear magnetic resonance (NMR), characteristic signals include aromatic protons (δ 6–8 ppm for H-6 and H-8 in the A-ring), prenyl methyl groups (δ 1.6–1.8 ppm), and carbonyl carbons (δ 170–180 ppm). Mass spectrometry typically displays a molecular ion peak at m/z 420.4 [M + H]^+^, with fragmentation patterns indicative of prenyl group loss (De Souza et al., 2000; Tan et al., 2025; Tan et al., 2025).

### 3.4 Physicochemical properties

Morusin chemical formula is C_25_H_24_O_6_, with a molecular weight of 420.5 g/mol. It has a melting point ranging from 214 °C to 216 °C. It behaves with low water solubility due to lipophilic prenyl groups, soluble in organic solvents (e.g., DMSO, methanol). It is also sensitive to light and heat; it degrades under prolonged basic conditions (Agarwal et al., 2018).

### 3.5 Biosynthesis

**FIGURE 2:**
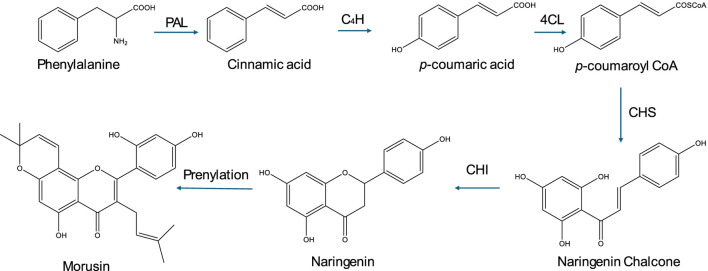
Biosynthesis of morusin. This figure illustrates the biosynthesis of morusin, highlighting its derivation from the phenylpropanoid pathway through flavonoid core formation followed by prenylation, which enhances its bioactivity, along with additional modifications such as hydroxylation and methylation.

Morusin biosynthesis follows the general flavonoid pathway with prenylation (Figure 2) (Liu S et al., 2022). Specifically, it begins with the phenylpropanoid pathway, which converts phenylalanine to p-coumaroyl-CoA. Next, flavonoid core formation occurs as chalcone synthase and chalcone isomerase generate naringenin. This is followed by prenylation, where isoprene units are added to the flavonoid backbone via prenyltransferases, leading to enhanced bioactivity. Additionally, several modification steps, including hydroxylation, methylation, and glycosylation, may occur, though morusin is typically non-glycosylated.

### 3.6 Quantification and standardization

Quantification and identification of morusin have been reported by several analytical methods. In flavonoid assays, method validation generally includes linear ranges of 0.1–50 μg/mL, recovery rates exceeding 90%, and limits of detection and quantification below 0.05 μg/mL (Pang et al., 2019). In HPLC/UPLC analyses, reverse-phase C18 columns coupled with UV detection at 280 nm are commonly used, with retention times generally falling between 15 and 25 min, depending on the system. LC-MS/MS provides exceptional sensitivity and specificity, making it a powerful analytical tool for detecting and quantifying morusin and its metabolites even at trace levels within complex matrices like plant extracts and biological fluids (Shi et al., 2016; Liu et al., 2019). Moreover, a recent study conducted an LC-MS/MS stable isotope dilution assay to quantify morusin in mulberry samples (Schnurr et al., 2025).

## 4 Pharmacological activities

### 4.1 Anti-inflammatory activity

It has been reported that elevated levels of inducible nitric oxide synthase (iNOS) and cyclooxygenase-2 (COX-2) are closely associated with inflammatory signaling (Saleron et al., 2002; Uchida, 2008). Nitric oxide (NO) is generated from the amino acid arginine via nitric oxide synthase and contributes to tissue injury at sites of inflammation. Meanwhile, COX-2 catalyzes the conversion of arachidonic acid to prostaglandins, such as PGE2, which are implicated in chronic inflammation and oxidative stress (Uchida, 2008). Morusin has been shown to reduce lipopolysaccharide (LPS)-induced nitrite and PGE2 production in a dose-dependent manner. Moreover, it significantly decreased LPS-induced COX-2 and iNOS levels in both *in vitro* rat macrophage RAW 264.7 cells and *in vivo* studies (Tseng et al., 2018).

Morusin also demonstrated therapeutic potential against *Mycoplasma pneumonia* by inhibiting Wnt/β-catenin activation and NF-κB (nuclear factor kappa-light-chain-enhancer of activated B cells) signaling pathways. It reduced pro-inflammatory cytokines, including interleukins; IL-6, IL-1β, and tumor necrosis factor-alpha (TNF-α), while enhancing anti-inflammatory IL-10 levels in mice lung tissue (Chen et al., 2019). As one of the main active phytochemicals in the traditional Chinese medicinal Shenjinhuoxue mixture, morusin contributed to the significant reduction of inflammatory mediators; Toll-like receptor 4 (TLR-4), IL-1 receptor-associated kinase 1, NF-κBp65, TNF-α, IL-6, IL-1β, receptor activator of NF-κB ligand (RANKL), and transient receptor potential vanilloid 1 (TRPV1) in the synovial and cartilage tissues of osteoarthritis (OA) rats following administration of the mixture (Ma et al., 2021).

Specifically, morusin attenuated inflammatory responses in OA by suppressing IL-1β-induced activation of TNF-α, IL-6, iNOS, and COX-2 in mouse chondrocytes *in vitro*. Additionally, it prevented articular cartilage degradation *in vivo* by modulating the NF-κB pathway, reducing the expression of ADAMTS5 and matrix metalloproteinases (MMPs) involved in extracellular matrix breakdown, and decreasing IL-1β-induced p65 phosphorylation and IκBα degradation (Jia et al., 2020). Yang et al. (2022) reported that morusin exerts protective effects against LPS-induced inflammation in ruminal epithelial cells by inhibiting protein kinase B (AKT) phosphorylation and NF-κB p65, through blockade of epidermal growth factor receptor (EGFR) signaling. Pretreatment with morusin (50 μg/mL for 12 h) significantly reduced pro-inflammatory factors, including TNF-α, cluster of differentiation 40 (CD40), IL-6, and CCL20, in LPS-stimulated ruminal epithelial cells (Yang et al., 2022).

Morusin may also serve as a potential anti-inflammatory agent for allergic and inflammatory skin disorders, such as atopic dermatitis. It inhibited RANTES/CCL5 (a chemokine that attracts various immune cells to sites of inflammation) and thymus- and activation-regulated chemokine secretion by blocking STAT1 (Signal Transducer and Activator of Transcription 1) and NF-κB p65 phosphorylation in TNF-α and interferon-stimulated HaCaT keratinocytes (Jin et al., 2019). In a 2,4,6-trinitrobenzene sulfonic acid (TNBS)-induced colitis model in rats, morusin exhibited potential for treating chronic inflammatory diseases. Its effects included the reduction of transforming growth factor-β1 (TGF-β1), a profibrogenic cytokine involved in inflammation and wound healing, decreased activity of matrix metalloproteinases two and 9 (MMP-2 and MMP-9), and a modest reduction in IL-1β levels (Vochyanova et al., 2017).

### 4.2 Antioxidant activity

Morusin was highlighted for its outstanding antioxidant activity over the other flavonoids as a result of its special chemical and bioactive characteristics (Choi et al., 2020). You and Kim (2019) declared that morusin could exert a neuroprotective effect against high glucose-induced oxidative stress in PC12 cells through inhibiting oxidative stress and intracellular reactive oxygen species (ROS) overproduction. Morusin decreased the levels of lipid peroxidation marker, the malondialdehyde (MDA), as well as significantly elevated the levels of the antioxidants glutathione (GSH) and superoxide dismutase (SOD) in high glucose-induced oxidative damage in PC12 cells (You and Kim, 2019).

Interestingly, the pathophysiology of cell senescence usually includes oxidative stress (Wiley et al., 2016). Nrf2, a transcription factor, plays a pivotal role in regulating genes responsible for antiaging proteins (Peng et al., 2022). Morusin significantly inhibited osteogenic medium induced reactive oxygen species (ROS) in senescence of aortic valve interstitial cells (VICs) by increasing the levels of Nrf2, hemeoxygenase-1 (HMXO-1), and NAD(P)H:quinone oxidoreductase 1 (NQO1) which are known for their antioxidant activities (Liu et al., 2024). On contrary, morusin exhibited no antioxidant effect in the cellular antioxidant assay that was employed for direct scavenging assays (Treml et al., 2021).

### 4.3 Central nervous system (CNS) activity

Morusin is a bioactive natural compound with growing recognition for its neuroprotective and neurotherapeutic potential. As research on neurodegenerative and neuropsychiatric disorders advances, interest in plant-derived therapeutics like morusin has intensified. Preclinical studies suggest that morusin exerts beneficial effects in the CNS through antioxidant, anti-inflammatory, anti-apoptotic, and neurotrophic mechanisms. With the global prevalence of CNS disorders including Alzheimer’s disease, Parkinson’s disease, epilepsy, stroke, and depression morusin emerges as a promising natural candidate for neurological interventions (Batiha et al., 2023; Choi et al., 2020). This section summarizes its pharmacological actions and supporting experimental evidence.

Neurons are highly metabolically active and particularly susceptible to oxidative stress due to elevated oxygen consumption and limited regenerative capacity. Accumulation of reactive oxygen species (ROS) contributes to neuronal death, synaptic dysfunction, and glial activation. Morusin mitigates oxidative stress by directly scavenging ROS and upregulating endogenous antioxidant defenses. Mechanistically, it activates the nuclear factor erythroid 2-related factor 2/antioxidant response element (Nrf2/ARE) signaling pathway, promoting transcription of antioxidant genes, including superoxide dismutase (SOD1, and SOD2), catalase, and glutathione (GPx). These effects have been confirmed in models of neurotoxicity and age-related stress, highlighting morusin’s role in preserving neuronal integrity (Cásedas et al., 2024; Hassanein et al., 2024).

Chronic neuroinflammation drives many CNS disorders, exacerbating neuronal injury and cognitive decline. Morusin reduces activation of microglia and astrocytes, key mediators of CNS inflammation. It inhibits NF-κB and mitogen-activated protein kinase (p38 MAPK) pathways, decreasing the transcription of pro-inflammatory cytokines such as IL-1β, IL-6, TNF-α, and monocyte chemoattractant protein-1 (MCP-1). Additionally, morusin downregulates iNOS and COX-2, limiting neuroinflammatory stress. Collectively, these actions foster a neuroprotective microenvironment and reduce glial scar formation (You et al., 2022; Liu et al., 2023).

Morusin also exerts strong anti-apoptotic effects, supporting neuronal survival under stress. It modulates mitochondrial apoptotic pathways by enhancing the expression of protective proteins while suppressing pro-apoptotic factors, stabilizing mitochondrial membranes, and preventing the release of apoptosis-triggering elements. In neuronal cultures and animal models of brain injury, morusin reduces apoptosis and preserves neuron viability in hippocampal and cortical regions (Choi et al., 2020; Panek-Krzyśko and Stompor-Gorący, 2021).

Moreover, mitochondria are central to energy metabolism and apoptosis regulation. Morusin maintains mitochondrial integrity by preserving membrane potential, reducing oxidative mitochondrial damage, and inhibiting the mitochondrial permeability transition pore (mPTP). These effects support adenosine triphosphate (ATP) production and limit mitochondrial-mediated neuronal apoptosis, particularly in conditions such as stroke and neurodegeneration (Cásedas et al., 2024; You and Kim, 2019; Cásedas et al., 2024).

Cognitive impairment is a hallmark of many neurological and psychiatric disorders. Experimental studies demonstrate that morusin improves spatial memory, learning, and recognition memory in rodent models. This effect may result from acetylcholinesterase inhibition, which increases synaptic acetylcholine, and modulation of glutamatergic transmission, enhancing hippocampal long-term potentiation (LTP), a key indicator of synaptic plasticity (Gupta et al., 2014; Gupta et al., 2014; Wang F et al., 2017).

In Alzheimer’s Disease (AD) models, morusin inhibits beta-secretase activity, reducing amyloid-beta (Aβ) plaque formation, and impairs tau phosphorylation, which is linked to neurofibrillary tangle formation and neuronal dysfunction. Its combined anti-amyloidogenic, anti-inflammatory, and antioxidant properties make it a promising candidate for disease-modifying therapy (Gupta et al., 2017; Gupta et al., 2017; Panek-Krzyśko and Stompor-Gorący, 2021).

Morusin protects dopaminergic neurons in the substantia nigra by mitigating oxidative stress and neuroinflammation as drug candidate for Parkinson’s disease (PD). It also inhibits monoamine oxidase-B (MAO-B) activity, preserving dopamine levels and improving motor function. Animal studies demonstrate enhanced locomotor activity and reduced bradykinesia following morusin treatment (Siddique et al., 2023).

Morusin exhibits anticonvulsant activity, potentially by enhancing gamma-aminobutyric acid (GABA)ergic signaling and inhibiting excitatory glutamatergic pathways in epilepsy. It reduces seizure frequency and duration in chemically induced epilepsy models, while also protecting neurons from oxidative damage (Rayam et al., 2019; Zehra et al., 2021).

Behavioral studies reveal that morusin possesses antidepressant and anxiolytic effects. It increases brain levels of monoamines such as serotonin and dopamine and upregulates hippocampal brain-derived neurotrophic factor (BDNF), a critical factor for mood regulation. These findings support its potential utility in major depressive disorder, anxiety disorders, and stress-related conditions (Zafar et al., 2013; Shahana and Nikalje, 2019).

### 4.4 Cardiovascular (CVS) activity

Morusin, traditionally known for its anticancer, anti-inflammatory, and antimicrobial effects, is now increasingly explored for cardiovascular applications. With cardiovascular diseases continuing to pose a major global health burden, recent studies have highlighted morusin’s ability to improve endothelial function, reduce oxidative stress, modulate inflammation, regulate lipid metabolism, and limit cardiac remodeling. This section explores the mechanisms underlying its potential in cardiovascular therapy (Liu et al., 2024).

Endothelial dysfunction is a critical early event in the pathogenesis of several cardiovascular conditions, including hypertension and atherosclerosis. This dysfunction often involves decreased nitric oxide (NO) production and bioavailability. Morusin has demonstrated the capacity to restore endothelial function by upregulating endothelial nitric oxide synthase (eNOS), thereby enhancing NO production. This effect contributes to improved vasodilation, reduced vascular resistance, and better regulation of blood pressure. Additionally, by maintaining NO levels, morusin may reduce platelet aggregation and leukocyte adhesion, processes that are central to vascular inflammation and thrombosis (Jin et al., 2025; Qi et al., 2025).

Oxidative stress is a central contributor to the development and progression of CVDs. Morusin exhibits strong antioxidant properties by both directly scavenging ROS and enhancing endogenous antioxidant defenses. It reduces lipid peroxidation and ROS levels, as demonstrated in various *in vitro* and *in vivo* studies. Concurrently, morusin activates the Nrf2 signaling pathway, leading to the upregulation of key antioxidant enzymes such as SOD, catalase, and glutathione peroxidase (GPx). This dual mechanism underscores morusin’s therapeutic potential in protecting against oxidative stress-induced cardiovascular injury (Zoofishan et al., 2018; Martins et al., 2021).

Chronic inflammation plays a pivotal role in vascular dysfunction and atherosclerosis. Morusin helps counteract this process through its anti-inflammatory effects, primarily by inhibiting the NF-κB signaling pathway. This inhibition suppresses the production of key pro-inflammatory cytokines such as TNF-α, IL-6, and IL-1β, thereby reducing vascular inflammation and promoting vascular health (Qi et al., 2025). Additionally, Morusin modulates immune cell behavior, particularly that of macrophages, which are central to atherosclerotic plaque development. It reduces macrophage recruitment and foam cell formation by downregulating scavenger receptor expression and lipid uptake pathways. These combined effects contribute to reduced plaque formation, stabilization of existing plaques, and a lower risk of plaque rupture and cardiovascular events (Jia et al., 2020).

Dyslipidemia is a significant risk factor for cardiovascular diseases, and morusin has demonstrated lipid-lowering effects in experimental models. It reduces levels of total cholesterol, low-density lipoprotein (LDL), and triglycerides, while simultaneously increasing high-density lipoprotein (HDL) levels, which are protective against atherosclerosis (Kim et al., 2022). In addition to improving circulating lipid profiles, Morusin interferes with lipid synthesis by downregulating key transcription factors and enzymes like fatty acid synthase (FAS). This suppression of endogenous lipid production further limits lipid accumulation in arterial walls, contributing to both the prevention and management of cardiovascular diseases and supporting overall metabolic health (Lee et al., 2018; Kadam et al., 2019; Nwakiban Atchan et al., 2022).

### 4.5 Hepatoprotective activity

Recent research has highlighted morusin’s hepatoprotective potential through its ability to shield the liver from toxin-induced damage. Morusin’s liver-protective effects are attributed to its capacity to combat oxidative stress and inflammation, inhibit apoptosis, and regulate liver enzymes, ultimately improving liver function and reducing damage (Hogade et al., 2010). Morusin’s antioxidant effect protects the body from damage by neutralizing free radicals, thanks to its free hydroxyl groups. This protection is achieved through several mechanisms, including neutralizing reactive oxygen species, binding to transition metals to prevent hydroxyl radical formation, shielding other antioxidants from oxidation, and preventing lipoprotein oxidation, ultimately safeguarding cells from oxidative stress and damage (Choi et al., 2020).

Morusin has been shown to inhibit the activation of nuclear factor-kappa B (NF-κB), a transcription factor that plays a key role in inflammation and immune responses. By suppressing NF-κB activation, morusin may help reduce inflammation and cell proliferation, contributing to its potential therapeutic benefits (Alharbi et al., 2022). Furthermore, morusin inhibited the upregulation of cyclooxygenase-2 (COX-2), which may be regulated by NF-κB. This inhibition can lead to decreased expression of pro-inflammatory genes, which may help alleviate various hepatic inflammatory conditions (Hafeez et al., 2023). Several studies showed that morusin demonstrates promise in treating various liver diseases. It may help slow the progression of liver cirrhosis by reducing inflammation and fibrosis. Additionally, morusin may alleviate hepatitis by minimizing liver inflammation and damage. Moreover, its potential anti-cancer properties make it a candidate for liver cancer treatment (Yin et al., 2018; Zhang et al., 2023).

### 4.6 Anti-diabetic activity

Morusin shows promise as a diabetes treatment due to its multifaceted anti-diabetic effects, but additional research is needed to unlock its full therapeutic potential, determine optimal dosing, and explore interactions with other diabetes medications (Foong et al., 2024).

Morusin’s anti-diabetic properties are attributed to several key mechanisms, including improved insulin sensitivity through enhanced glucose uptake in skeletal muscle cells and adipocytes, regulation of glucose metabolism by modulating enzymes like glucokinase and glucose-6-phosphatase, antioxidant effects that reduce oxidative stress, and inhibition of pro-inflammatory signaling pathways that contribute to insulin resistance and diabetic complications (Kawser Hossain et al., 2016).

Morusin shows potential for managing diabetes by increasing insulin sensitivity through enhanced glucose uptake, and safeguarding pancreatic β-cells, thereby supporting insulin production (Lv et al., 2022). Morusin improves insulin function by enhancing insulin sensitivity, promoting glucose uptake in skeletal muscle cells and adipocytes, and activating key proteins involved in insulin signaling. It also protects pancreatic β-cells from damage and apoptosis, preserving insulin secretion. Additionally, morusin regulates glucose metabolism by modulating enzymes involved in glucose production and utilization, collectively contributing to its potential as an anti-diabetic agent (Paudel et al., 2018).

Additionally, morusin exhibits multifaceted therapeutic effects, including inhibition of α-glucosidase to delay intestinal carbohydrate digestion, amelioration of skeletal muscle insulin resistance, reduction of lipid accumulation and hepatic steatosis, improvement of insulin signaling, and mitigation of oxidative stress, collectively contributing to its potential in managing diabetes and related metabolic disorders (Hsu et al., 2022).

Several studies have shown that mulberry extracts rich in morusin have promising anti-diabetic effects. In diabetic rats, mulberry leaf extract lowered blood glucose levels, reduced oxidative stress, and improved insulin levels. The extract also blocked glucose absorption and slowed its transit (Singab et al., 2005; Naowaboot et al., 2009). Additionally, other studies demonstrated that morusin regenerated pancreatic β-cells, restored normal pancreatic function, and balanced pancreatic weight. These findings suggest that morusin may be a beneficial supplement for managing diabetes (Mohammadi and Naik, 2012).

### 4.7 Bone repair activity

**FIGURE 3:**
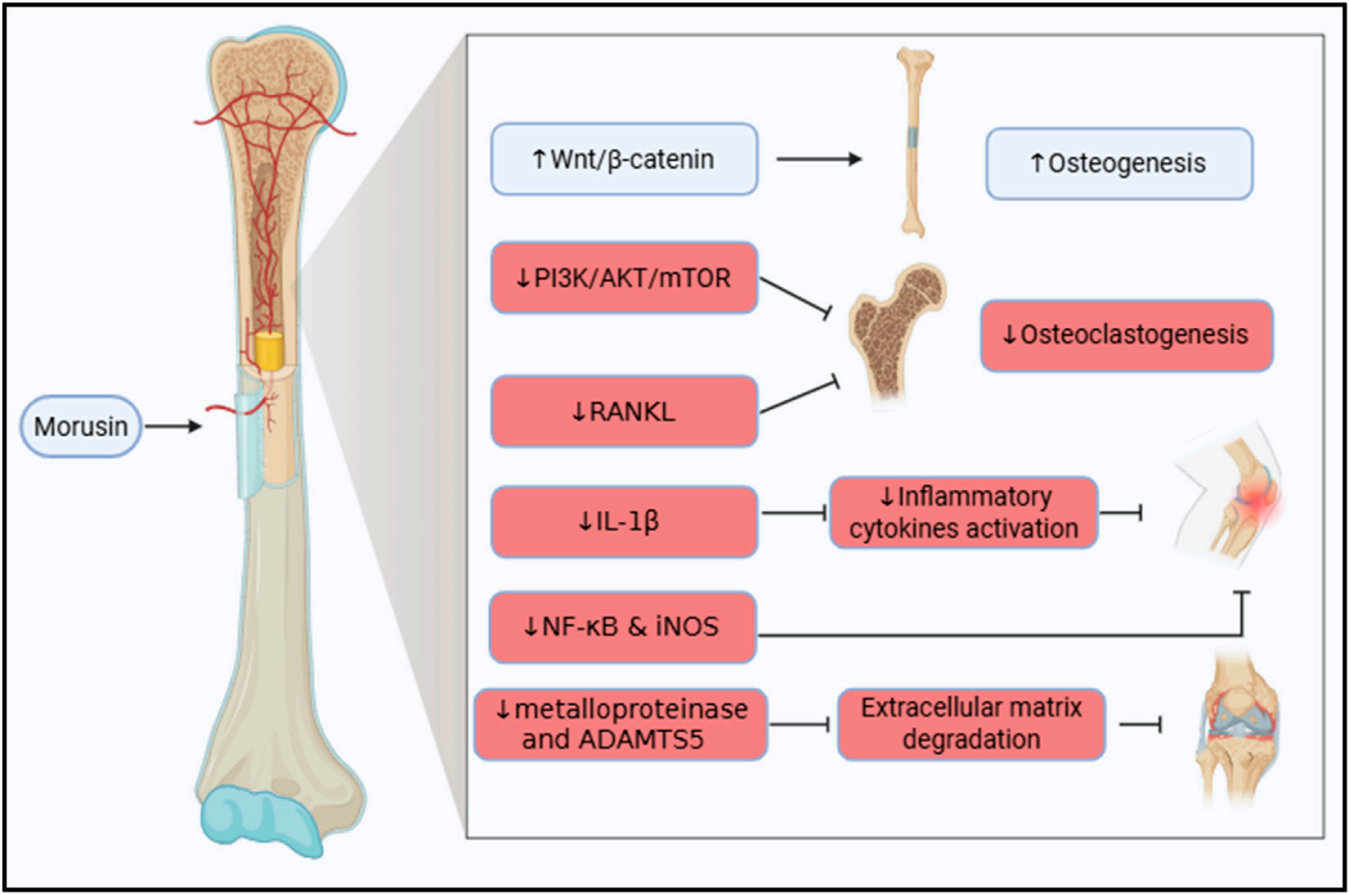
Illustration of the therapeutic molecular pathways exhibited by morusin in bone disorders, highlighting its dual role in promoting osteogenesis by stimulating Wnt/β-catenin signaling and inhibiting osteoclastogenesis and inflammation via suppression of PI3K/AKT/mTOR and NF-κB pathways, thereby supporting bone formation and reducing bone degradation.

Bone health is crucial for overall wellbeing, and its deterioration can lead to mobility impairments, significantly affecting quality of life (Ferrucci et al., 2014). In recent years, ethnopharmacology has gained attention as a potential strategy for enhancing bone remodelling and repair. This section focuses on the therapeutic potential of morusin in bone disorders, particularly osteoporosis and osteoarthritis. The proposed mechanisms of morusin’s action in bone disorders are summarized in Figure 3.

Morusin has been recognized for its osteogenic potential. It inhibits the differentiation of bone-resorbing cells, the osteoclasts (Zhang et al., 2024). In addition, the mechanism was studied by Chen et al. both *in vivo* and *in vitro*. Bone marrow mesenchymal stem cells (BMSCs) were harvested and treated with increasing concentrations of morusin. Interestingly, morusin was found to enhance proliferation as well as the differentiation of BMSCs into osteoblasts, thus boosting osteogenesis. Mainly, the mechanism by which morusin acts as an osteogenic agent is via stimulating the Wnt/β-catenin signaling pathway. This activity was not only observed *in vitro* but also *in vivo* in ovariectomized rats (Chen et al., 2021).

In breast cancer, morusin was tested as an osteoprotective agent. Regrettably, in breast cancer and in osteosarcoma, PI3K/AKT/mTOR signaling is highly activated to promote tumor growth and invasion (Zhang et al., 2020; Li et al., 2023). The PI3K/AKT/mTOR signaling pathway is also accused of promoting osteoclastogenisis, thus, resulting in bone resorption (Zhang et al., 2024). Fortunately, morusin has the capability to inhibit the PI3K/AKT/mTOR signaling. As a result, it proficiently contribute to the prevention of, not only osteosarcoma and breast cancer, but also to the prevention of breast cancer-induced osteolysis (Zhang et al., 2020; Zhang et al., 2024). Additionally, Jin et al. corroborated that morusin succeeded in hampering the RANKL-induced osteoclastogenesis in an ovariectomy-induced osteoporosis model (Jin et al., 2024).

Osteoarthritis (OA) is an inflammatory bone disorder where IL-1β plays a central role in initiating inflammatory cascades. Morusin has been shown to attenuate IL-1β-induced activation of pro-inflammatory cytokines (Choi et al., 2020). Additionally, it modulates the expression of NF-κB and iNOS, key mediators of inflammation in OA (Jia et al., 2020).

Beyond inflammation, extracellular matrix (ECM) degradation, driven by enzymes such as metalloproteinases and ADAMTS5, contributes to cartilage destruction. Administration of morusin reduces the expression of these enzymes, preserving bone density and ECM integrity. Collectively, these effects demonstrate morusin’s chondroprotective and anti-inflammatory properties, highlighting its potential as a therapeutic agent in OA (Jia et al., 2020).

### 4.8 Analgesic and antinociceptive activity

Morusin’s analgesic effect could be compared to the commonly used acetylsalicylic acid, offering a safer option (de Souza et al., 2000; Hafeez et al., 2023). This beneficial effect could be credited, at least in part, to manipulating metabotropic glutamate receptor 1, as shown by (Wang F et al., 2017) Besides the glutamate receptor, morusin demonstrated the ability to block cyclooxygenase (COX) enzymes needed for the generation of pain mediators (Chi et al., 2001; Panek-Krzysko and Stompor-Goracy, 2021). Surprisingly, morusin showed a preference for COX-2 inhibition over COX-1 in aspirin-pretreated cells (Chi et al., 2001).

### 4.9 Anticancer and immunomodulatory activities

Cancer is a major worldwide health concern and the leading cause of death, second only to cardiovascular disease. Hence, drug researchers are increasingly focusing on generating safe, low-toxic, and effective anticancer drugs (Hassan et al., 2025). Morusin is a natural substance derived from the bark of Morus Alba, a mulberry tree. Morusin has shown anti-tumor activity in a variety of cancers, including breast, prostate, gastric, hepatocarcinoma, glioblastoma, and pancreatic cancer. Morusin’s potential as an alternate therapy for resistant cancers is being investigated in animal models with the goal of moving forward with human trials. Morusin has been proven in laboratory studies to hinder the development of breast, ovarian, and colon cancers. There is evidence that morusin can induce cancer cells to commit suicide through a process known as apoptosis. Recent studies demonstrated that morusin can inhibit the activation of NF-κB and activators of STAT-3 in cancer cells, including prostate, pancreatic, and liver (Agarwal et al., 2019).

Although morusin has anticancer characteristics, the particular mechanisms by which it operates are unknown; nonetheless, it is expected to block important signaling pathways that promote tumor growth and survival. Morusin displayed high cytotoxicity in Huh7 and Hep3B cells using the MTT (a cell viability test) and CCK-8 tests, and it decreased the number of colonies in these cells, showing anticancer activity. Similarly, by disrupting the IL6/STAT3 signaling pathway, morusin promoted apoptosis and inhibited angiogenesis in hepatocellular carcinoma cells (Cho et al., 2021).

Morusin inhibited renal cell carcinoma (RCC) cell growth and migration in a dose and time-dependent manner. Morusin has been shown to promote apoptosis and cell cycle arrest in the G1 phase by increasing the expression of Bax and cleaved-caspase three while suppressing the expression of Bcl-2, CDK4, CDK6, and Cyclin D1. These findings suggested that morusin might be an effective therapy option for RCC (Yang C et al., 2020). Morusin was discovered to inhibit the initiation of colorectal cancer spheroid formation as well as the proliferation of colorectal HCT116 sphere cells. Morusin also decreased the expression of two stemness markers, Oct4 and Nanog. The study demonstrated possible molecular routes for morusin’s impacts on colon cancer stem cells (CCSCs) (Zhou et al., 2021). A recent study found that morusin can block human NPC HONE-1, NPC-39, and NPC-BM cells from migrating and invading. These studies demonstrated that morusin is helpful in inhibiting nasopharyngeal cancer (NPC) invasion and metastasis (Huang et al., 2020). According to a recent study, morusin may promote necrosis and autophagy in breast cancer cells, as well as other types of cell death. The study demonstrated that morusin, which inhibits the anti-apoptotic protein survivin, might be used as an anti-cancer medicine to treat breast cancer (Kang et al., 2017). Morusin demonstrated that it disrupted the proto-oncogene tyrosine-protein kinase/Janus kinase 2/signal transducer and activator of transcription 3 (SRC/JAK2/STAT3) signaling pathway by blocking their phosphorylation, resulting in the loss of STAT3 nuclear accumulation and transcriptional activity. These findings suggest that morusin may be an effective anti-cancer therapy for prostate cancer since it induces apoptosis in prostate cancer cells by inhibiting STAT3 and increasing SHP1 (Lim et al., 2014). In one investigation, various doses of morusin were administered to the gastric cancer cell lines MKN45 and SGC7901. According to the results of tests employing MTT and bromodeoxyuridine (BrdU), morusin was able to suppress the development of gastric cancer cells in a dose-dependent manner (Wang Y et al., 2017).

**FIGURE 4:**
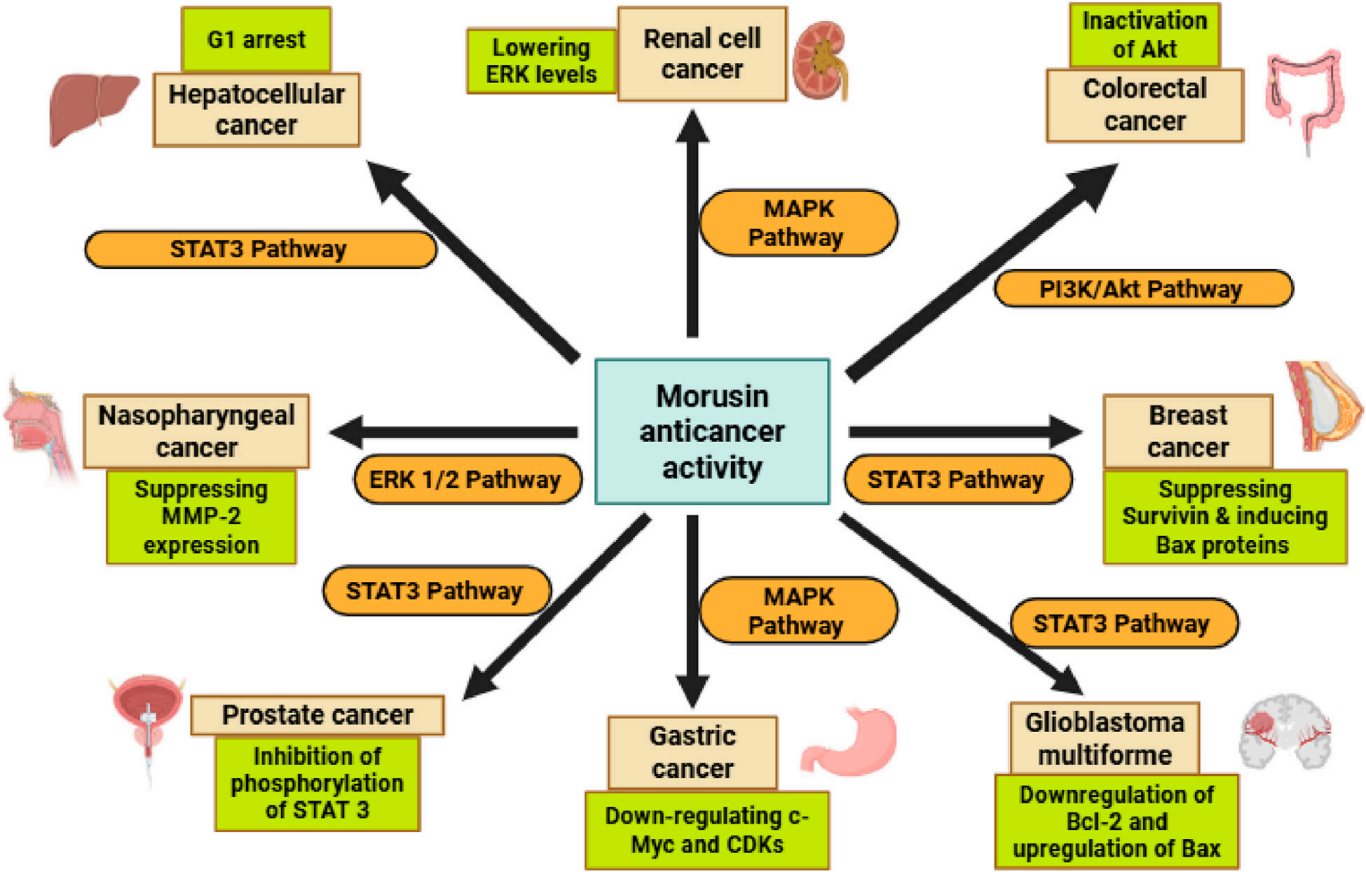
The scope of morusin anticancer activity, through its modulation of key signaling pathways and proteins, including STAT3, MAPK, Bcl-2, CDK, C-Myc, BAX, and MMP, which collectively regulate cell proliferation, apoptosis, and metastasis. Abbreviations: STAT3, Signal transducers and activators of transcription 3, MAPK, Mitogen-activated protein kinase, Bcl-2, B-cell lymphoma 2, CDK, Cyclin-dependent kinases, C-Myc, Cellular myelocytomatosis, BAX, Bcl-2-associated X protein, MMP, matrix metalloproteinase.

Morusin has a strong growth-inhibiting impact on human GBM cancer stem cells (GSCs) both *in vitro* and *in vivo*, and its mechanism of action may include GSC stemness reduction, GSC conversion into adipocytes, and death induction. More research is needed since it might be the first therapeutic medicine to directly target GSCs for the treatment and/or prevention of human glioblastoma multiforme (GBM) (Guo et al., 2016). Figure 4 is a summarized schematic illustration that clearly displays morusin’s actions against different cancer types.

### 4.10 Nephroprotective activity

**FIGURE 5:**
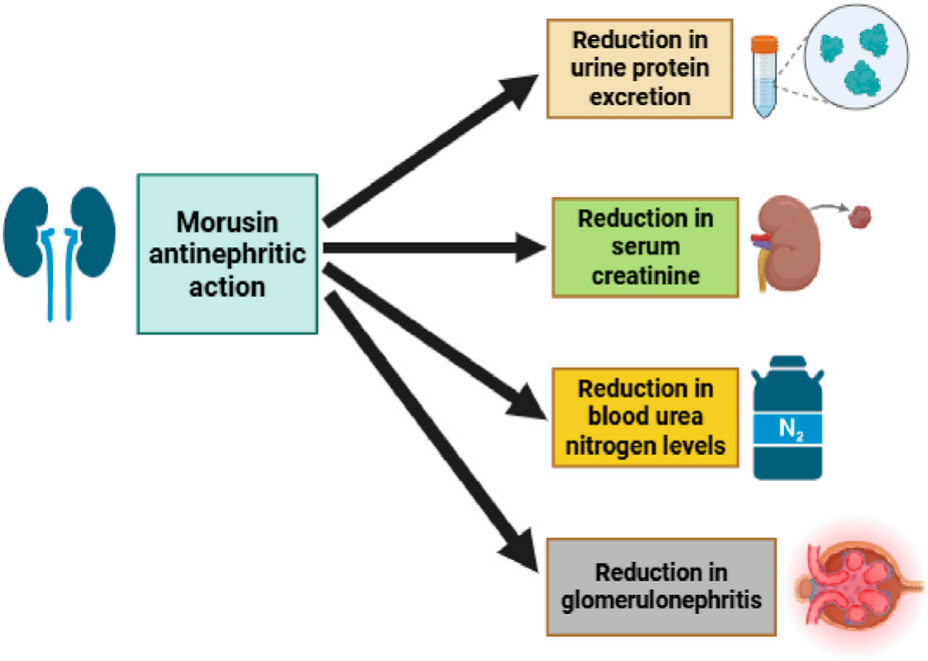
Morusin antinephritic action, highlighting its ability to reduce urinary protein excretion, serum creatinine, blood urea nitrogen, and total cholesterol levels, thereby ameliorating kidney dysfunction in glomerular disease models.

A recent study investigated the antinephritic properties of morusin and its analogs, focusing on their effects in a mouse model of glomerular disease. The evaluation included key renal function parameters such as urinary protein excretion, total cholesterol, serum creatinine, and blood urea nitrogen. Results demonstrated that morusin significantly reduced proteinuria, serum creatinine, and blood urea nitrogen levels, as confirmed by Electron Spin Resonance (ESR) spectroscopy (Figure 5). These findings indicate that morusin may exert a protective effect against renal dysfunction. In line with these results, earlier *in vivo* studies also reported that morusin provided beneficial effects in chemically induced glomerulonephritis models, further supporting its potential as a nephroprotective agent (Fukai et al., 2003). Taken together, these data suggest that morusin’s therapeutic activity may extend beyond anticancer and metabolic disorders, highlighting its promise in the management of kidney-related diseases.

### 4.11 Antiviral activity

Pseudorabies Virus (PRV) is a well-characterized member of the α-herpesvirus subfamily (Zheng et al., 2022). Pigs are its natural hosts, and PRV can cause a range of infections affecting the respiratory, nervous, and reproductive systems. Alarmingly, PRV can also infect humans, resulting in severe and potentially life-threatening conditions such as encephalitis and visual impairments, posing a significant public health concern (Yang H et al., 2020). Mulberry leaves (*M. alba* L.; MLE) are widely cultivated in Asia and have been used in traditional medicine for thousands of years. Recent research has investigated the antiviral potential of MLE against PRV (Zhang, et al., 2025). The study demonstrated that MLE markedly inhibits PRV replication, with co-treatment of MLE and PRV showing the most significant inhibitory effect. The antiviral activity primarily targets the early stages of infection, including virus adsorption and entry, with inhibition of the entry stage being more pronounced than adsorption. One key constituent, Kuwanon X, has been shown to prevent adsorption and entry of herpes simplex virus type 1 (HSV-1) (Liu Y et al., 2022). Additionally, MLE contains flavonoids such as kaempferol, quercetin, and resveratrol. Kaempferol has been reported to inhibit PRV replication in organs including the spleen, lungs, heart, and kidneys (Li et al., 2023), while quercetin effectively inhibits infection by multiple PRV strains (Dhiman et al., 2020).

Herpes Simplex Virus Type 1 (HSV-1) infects approximately 85% of adults, causing diseases such as oral-labial lesions, keratitis, genital infections, and encephalitis (Hassan et al., 2017; Crimi et al., 2019). Following primary infection, HSV-1 establishes latency in the trigeminal ganglion, contributing to long-term neurologic effects (Arduino and Porter, 2008). Diagnosis increases with age, as asymptomatic carriers may remain undetected. HSV-1-induced tissue damage is mediated by excessive reactive oxygen species (ROS) production and oxidative stress, which play a major role in viral pathogenesis (Hu et al., 2011). While ROS are beneficial in eradicating pathogens (Gonzalez-Dosal et al., 2011), they also promote viral replication and trigger cellular apoptosis via HSV-1 protein ICP27 (Kim et al., 2008; Hu et al., 2011). Inhibition of ROS can therefore prevent viral replication and reduce cell death (Chen et al., 2015). Excessive ROS also induces mitochondrial fragmentation, disrupting mitochondrial dynamics and creating a vicious cycle of ROS overproduction and reduced ATP synthesis, ultimately leading to cell death (Zorov et al., 2014; Guo et al., 2016). MLE treatment significantly decreases mitochondrial and cellular ROS in Vero cells in a dose-dependent manner, thereby preventing host cell death (Kim et al., 2022).

**FIGURE 6:**
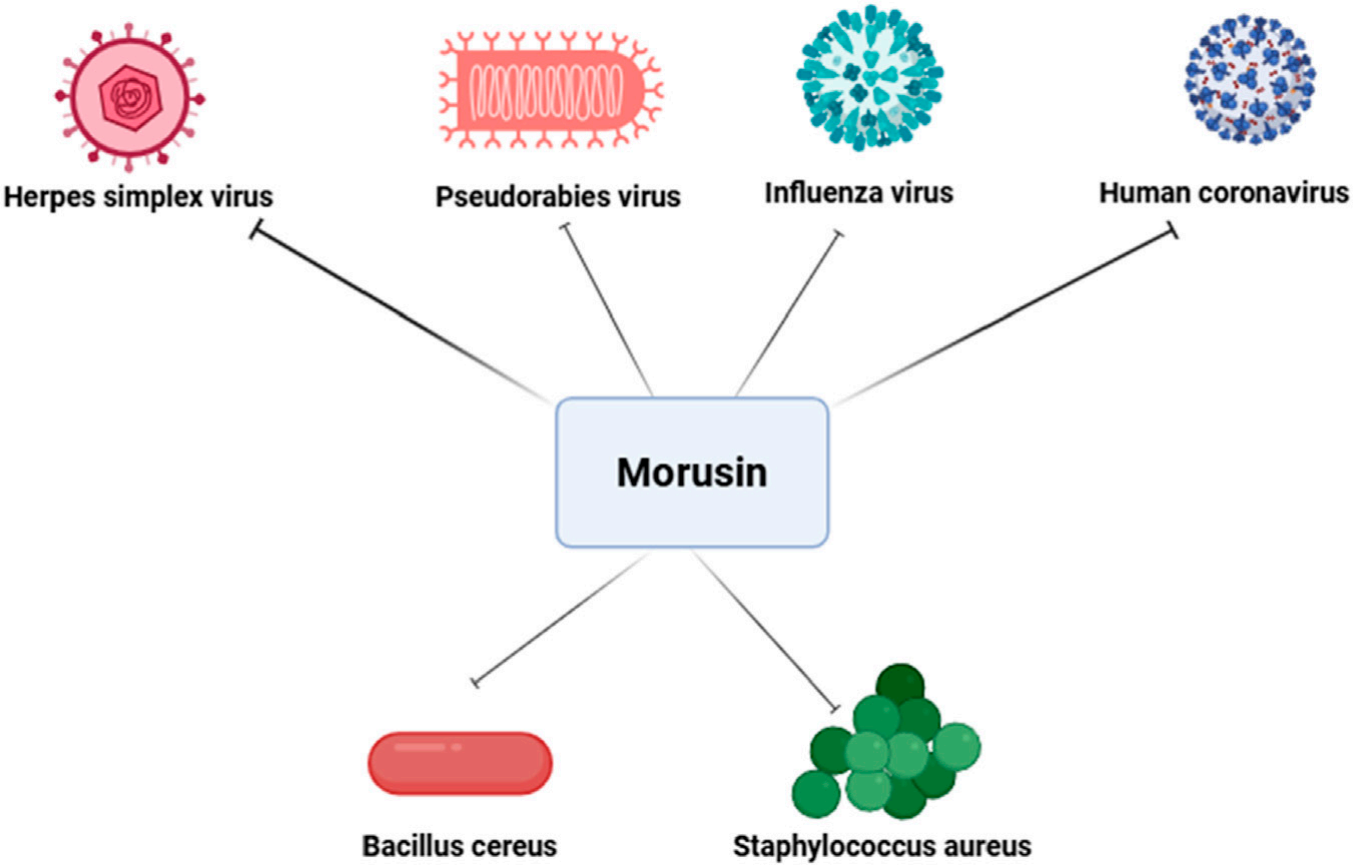
Antiviral and antibacterial effects of morusin. This figure demonstrate its ability to inhibit viral replication and entry in pathogens such as PRV, HSV-1, influenza virus, and human coronavirus, while also disrupting bacterial cell membrane integrity and biosynthesis pathways in Gram-positive pathogens like *Staphylococcus aureus* and *Bacillus cereus*.

Influenza virus, a highly diverse pathogen subject to antigenic drift and shift, causes acute respiratory infections and seasonal epidemics, significantly impacting society, healthcare systems, and economies worldwide (Jennings et al., 2018; Kadam and Wilson, 2018). Influenza infection activates ROS and promotes oxidative stress, which facilitates viral replication. Severe infections particularly threaten children under five, the elderly, and immunocompromised individuals. Pre-treatment or co-treatment with MLE has shown robust antiviral effects against various influenza strains in a dose-dependent manner (Kim and Chung, 2018). The antiviral effect of MLE is linked to inhibition of viral surface protein attachment to host cell receptors. Moreover, MLE restores intracellular glutathione (GSH) levels and scavenges free radicals, counteracting influenza-induced redox imbalance and reducing viral replication (Di Sotto et al., 2018). During the COVID-19 pandemic, one of the most significant global health crises (Khalifa and Al Ramahi, 2024), mulberry leaf extract (MLE) was reported to display antiviral activity against human coronavirus (HCoV-229 E) (Thabti et al., 2020). These results underscore the potential of *Morus* species constituents as promising candidates for the development of novel antiviral therapeutics (Figure 6).

### 4.12 Antibacterial activity

Antibiotic Antibiotic treatment of chronic wounds remains a major clinical challenge, as bacteria frequently develop resistance when antibiotics are used inappropriately or for prolonged periods (Tchero et al., 2018). Patients with MRSA infections, for instance, face a 64% higher risk of mortality compared to those with drug-susceptible infections. One strategy to overcome resistance in bacterial strains is to combine natural compounds with antibiotics, thereby achieving synergistic antimicrobial effects (Cheesman et al., 2017). Among natural compounds, flavonoids have shown particular promise; their lipophilicity is considered a key factor driving activity against Gram-positive bacteria (Kushwaha et al., 2020). Phenolic compounds derived from mulberry leaf extract (MLE), both alone and in combination with conventional antibiotics, have demonstrated *in vitro* antibacterial activity and are being investigated as alternative approaches for wound healing and MRSA infection management (Čulenová et al., 2020; Zhang X et al., 2021).

The microbial landscape of wounds further emphasizes the need for new therapeutic options. Acute wounds are typically dominated by Gram-positive bacteria, whereas chronic wounds often harbor both Gram-positive species—such as *Staphylococcus aureus* and *Staphylococcus epidermidis*—and Gram-negative bacteria, including *Escherichia coli*, *Proteus mirabilis*, *Pseudomonas aeruginosa*, *Enterobacter* spp., and *Morganella* spp. (Maheswary et al., 2021; Škovranová et al., 2022). Phenolic compounds, including prenylated flavonoids, are particularly attractive because they not only possess antibacterial effects but also exhibit antioxidant and anti-inflammatory activities, which are highly beneficial for wound healing.

Beyond wound pathogens, morusin has demonstrated activity against *Bacillus cereus*, a Gram-positive, facultative anaerobic *bacillus* capable of contaminating the food chain through crops and causing foodborne illnesses such as diarrhea and vomiting, thereby posing a significant threat to food safety (Liao et al., 2024). A recent study revealed that morusin’s bactericidal mechanism against *S. aureus* and *Salmonella* involves disruption of cell membrane integrity and interference with phosphatidic acid biosynthesis, a critical pathway for phospholipid synthesis. Morusin treatment was also shown to alter the expression of genes linked to phosphatidic acid and fatty acid biosynthesis, further impairing bacterial viability (Mazimba et al., 2011; Pang et al., 2019). In addition to antibacterial activity, morusin has been reported to exhibit antifungal properties (Hafeez et al., 2023). While its antibacterial mechanisms are relatively well-characterized—such as membrane disruption and inhibition of phosphatidic acid biosynthesis—the precise antifungal mode of action remains less clear. The potential binding sites of morusin on fungal cell membranes and the pathways involved have not yet been fully elucidated, and existing studies have not sufficiently explained why morusin does not appear to easily induce drug resistance in fungi. Taken together, these findings highlight morusin as a promising natural compound with both antibacterial and antifungal potential. In the context of escalating antimicrobial (Khalifa et al., 2021) and antifungal resistance (Khalifa et al., 2022), morusin and related flavonoids may represent valuable candidates for the development of next-generation therapeutic strategies aimed at overcoming drug resistance and improving outcomes in chronic wound management, infectious diseases, and food safety (Figure 6).

## 5 Safety and toxicity profile

Current evidence on morusin’s safety is limited. Acute toxicity studies in rodents indicate a relatively high tolerance, with no mortality or severe clinical signs at doses up to 100 mg/kg; however, comprehensive biochemical and histopathological data are lacking (Hafeez et al., 2023). Long-term safety, including potential hepatic, renal, reproductive, or immunotoxic effects, remains unexplored. *In vitro* findings suggest selective cytotoxicity toward malignant cells (Choi et al., 2020), yet potential drug–drug interactions are plausible due to morusin’s inhibition of CYP3A4 and UGT1A1 (Shi et al., 2015; Liu et al., 2019). Limited reports of hepatic enzyme alterations at high doses highlight the need for careful dose extrapolation, especially given species differences in metabolism, with human CYP-mediated clearance being markedly slower than in rodents (Feng et al., 2022). Systematic toxicological studies are therefore essential to define a safe therapeutic window before advancing to clinical trials.

## 6 Clinical data

To date, no confirmed clinical trials or human studies on morusin have been reported, underscoring the need to better understand its therapeutic potential. A recent review emphasized that morusin’s development remains largely at the preclinical stage, with efficacy demonstrated mainly *in vitro* and in animal models (Hafeez et al., 2023). Morusin has shown strong activity across diverse disease models, particularly anticancer effects in various cancer types, but its translation to clinical testing is still unproven. Notably, morusin has exhibited antitumor activity in xenograft and rodent cancer models and has improved metabolic outcomes in diabetic rodents (Kang et al., 2025), reinforcing its potential clinical value (Feng et al., 2022).

Despite these findings, no human clinical trials have yet been initiated. As of 2025, no studies involving human subjects are registered in public databases (Hafeez et al., 2023; Panek-Krzyśko and Stompor-Gorący, 2021), and available evidence remains limited to scarce clinical observations. This represents a major gap in the literature and highlights the urgent need for clinical investigations. In the meantime, translational animal studies continue to provide important insights: the demonstrated antihyperglycemic and anti-inflammatory effects of morusin in diabetic rodents suggest potential applications in metabolic and inflammatory disorders (Araujo et al., 2015; Ramos et al., 2021; Azab, 2023). While preclinical data appear highly promising, critical information regarding safety, pharmacokinetics, and efficacy in humans is still lacking. Therefore, well-designed clinical trials are essential before morusin can be seriously considered as a therapeutic candidate.

## 7 Pharmacokinetic properties

Morusin, being a prenylated (isoprene-substituted) flavone with poor water solubility, significantly influences its ADME characteristics. Most pharmacokinetic data available to date are from animal models (rats); only a few *in vitro* experiments were conducted using human-derived systems (Panek-Krzyśko and Stompor-Gorący, 2021). Currently, only limited reports are available on the pharmacokinetics of morusin. However, new insights into its ADME characteristics are just beginning to emerge (Xiong et al., 2023).

### 7.1 Absorption and bioavailability

The physicochemical properties of morusin are a significant barrier to the oral absorption of this compound. Being a highly hydrophobic hydroxyflavone, it is almost insoluble in water and barely dissolves in gastrointestinal fluids (Panek-Krzyśko and Stompor-Gorący, 2021). As such, much of the morusin struggles to be absorbed orally; recent pharmacokinetic evaluation, for example, has estimated that the oral bioavailability is around ∼11.5% when tested in rats (Hafeez et al., 2023), thereby falling abysmally short of the 20% threshold generally regarded as the minimum for drug-likeness. This marginal oral bioavailability partially results from decreased gastrointestinal absorption and a highly extensive first-pass effect, as well as solubility-constrained transmembrane uptake (Agarwal et al., 2018). Plasma levels of morusin in rats given an extract containing this compound (mulberry root bark extract) are very low; e.g., maximum concentration (Cmax) peaked at ∼16.8 ng/mL post 2 g/kg dose of extract prepared from Mori Cortex-flavonoid content of ∼20 mg/kg in normal rats (Xiong et al., 2023). The best hope for better absorption of morusin might come from some innovative possibilities: a consequent study accounted for the apparent improvement in bioavailability of morusin with niosome-incorporated nanocarrier interaction (Agarwal et al., 2018). Nonetheless, peak levels reached after oral administration 1–2 h later are still very low, and absorption remains incomplete. Multiple concentration-time profiles are seen for morusin after oral administration (Deng et al., 2017). This phenomenon suggests an enterohepatic recycling mechanism in which, after initial absorption and metabolism, the conjugated metabolites are excreted through the bile and hydrolyzed in the gut to yield morusin (or active metabolites) for reabsorption. This enterohepatic circulation effectively extends the presence of morusin in circulation despite the limited absorption, thus resulting in a second plasma peak (Hou et al., 2018). Overall, oral absorption of morusin is modest. Therefore, enhancing bioavailability seems to be the way forward in future studies, either through increased solubility or a prodrug approach.

### 7.2 Distribution

Even before considering its tissue distribution, there is very little data available on morusin (Panek-Krzyśko and Stompor-Gorący, 2021). Given the lipophilicity of morusin, some degree of tissue distribution is expected, while at the same time it could bind to plasma proteins. There are no human data for the volume of distribution (Panek-Krzyśko and Stompor-Gorący, 2021). Studies in rats were mainly constrained to plasma pharmacokinetics rather than full tissue distribution profiles (Hou et al., 2018). To provide an idea, hypothetically, a prenylated flavonoid would possibly have a moderate volume distribution, potentially accumulating primarily in the liver and fatty tissues. It has been reported that morusin, after oral administration of ethylenediaminetetraacetic acid (EDTA), showed elevated concentrations; an individual observed high levels, in the microgram range, in well-perfused organs like the liver (being a site of metabolic activity) (Haywood and Glass, 2011; Di and Kerns, 2016). Hence, quantitative tissue distribution studies have not been accomplished so far (Panek-Krzyśko and Stompor-Gorący, 2021). Because of extensive first-pass metabolism, most of the circulating morusin would perhaps persist in one or more metabolized forms rather than as the parent compound itself. Most importantly, metabolites of morusin (glucuronides) are exceedingly polar and mostly circulate just between plasmatic compartments and excretory outlets. Statistically speaking, therefore, while no definitive tissue distribution work is at our disposal, one could imagine morusin being distributed in the liver (site of metabolism) but also covering some other tissues to some degree, although no evidence indicates significant tissue accumulation (Hou et al., 2018; Liu et al., 2019; Xiong et al., 2023). This is another basic line of study that should provide greater insight into morusin tissue disposition *in vivo*.

### 7.3 Metabolism (phase I and II)

Metabolism is the key pharmacokinetic process determining the fate of morusin. Once absorbed, morusin undergoes extensive first-pass metabolism in both the intestine and liver. Experimental evidence from rats and *in vitro* human systems indicates that Phase II conjugation (glucuronidation) is the dominant pathway (Hou et al., 2018), while Phase I oxidation plays only a minor role (Panek-Krzyśko and Stompor-Gorący, 2021).

#### 7.3.1 Glucuronidation

Morusin is rapidly conjugated with glucuronic acid by UDP-glucuronosyltransferase (UGT) enzymes. In a detailed rat study using portal vein-cannulation and LC-MS analysis, four monoglucuronide metabolites were identified: M-5-G, M-4′-G, M-2′-G, and M_II-2 (Hou et al., 2018). In plasma, three metabolites (M-5-G, M-4′-G, and M_II-2) were detected, whereas all four were present in bile and intestinal perfusates (Panek-Krzyśko and Stompor-Gorący, 2021). Among them, M-4′-G (the 4′-O-glucuronide) was the predominant circulating metabolite. Only trace levels of the parent morusin remain in systemic circulation.

Mechanistic *in vitro* studies with recombinant human UGTs identified UGT1A1, UGT1A3, UGT1A7, and UGT2B7 as the major isoforms catalyzing morusin glucuronidation (Shi et al., 2015). Kinetic analyses indicated substrate inhibition kinetics, where high concentrations of morusin slow metabolism due to enzyme saturation. The intrinsic clearance rate of morusin glucuronidation in human liver microsomes (∼130 mL/min/mg protein) was comparable to rats, suggesting similar metabolic efficiency across species (Hou et al., 2018). Additionally, intestinal UGTs contribute significantly, as glucuronides were detected in intestinal perfusate samples.

#### 7.3.2 Oxidative metabolism (phase I)

Compared with glucuronidation, oxidative metabolism via cytochrome (CYP) P450 enzymes is minor. Rat studies revealed only trace amounts of a single oxidative metabolite (M_I-1) in plasma, with glucuronides dominating bile and gut samples (Shi et al., 2016). In human liver microsomes, hydroxylation was the predominant Phase I reaction, generating up to six oxidative metabolites through hydroxylation, reduction, and dehydration (Liu et al., 2019).

Among cytochrome P450 (CYP) enzymes, CYP3A4 was identified as the principal isoform mediating morusin oxidation, while CYP2C9 and CYP1A2 contributed minimally (Liu et al., 2019; Haywood and Glass, 2011). The structure of morusin, a prenylflavone, also enables it to inhibit metabolic enzymes. At high concentrations (≥100 μM *in vitro*), morusin inhibited multiple CYPs (3A4, 1A2, 2C9) and UGTs, with IC_50_ values in the low micromolar range (Di and Kerns, 2016). While such concentrations may not be physiologically relevant, these findings suggest possible drug–herb interactions if morusin is co-administered with drugs metabolized by the same enzymes.

### 7.4 Metabolic pathways and enzyme involvement

Morusin metabolism involves both Phase I and Phase II processes, with Phase II glucuronide conjugation being the predominant pathway. The primary enzyme system responsible for morusin conjugation is the UGT pathway, specifically UGT1A1, UGT1A3, UGT1A7, and UGT2B7, which catalyze glucuronidation at multiple hydroxyl positions (Panek-Krzyśko and Stompor-Gorący, 2021). Among these, the 4′-O-glucuronide is identified as the major glucuronide metabolite, representing the bulk of circulating metabolites. While UGT-mediated clearance is usually a negligible route for most compounds, it plays a crucial role in the metabolism of morusin. Inhibition of glucuronidation results in a marked reduction in morusin clearance (Panek-Krzyśko and Stompor-Gorący, 2021).

By contrast, Phase I contributes only minimally to morusin metabolism. Human liver microsomes are considered a suitable system for evaluating these limited Phase I reactions. CYP enzymes play only a minor role, with CYP3A4 (and its rat analog CYP3A) identified as the principal isoform mediating oxidative bioconversion (Liu et al., 2019). Other cytochrome P450 isoforms, including CYP2C9, CYP2C19, and CYP1A2, exhibit negligible contributions *in vitro* (Panek-Krzyśko and Stompor-Gorący, 2021). Taken together, Phase I oxidation is far less prominent compared to the dominant Phase II glucuronidation pathways of morusin metabolism.

The prenylated structure of morusin confers inhibitory activity against drug-metabolizing enzymes. *In vitro* studies show that at micromolar concentrations, morusin competitively inhibits several CYP isoforms (CYP3A4, CYP1A2, CYP2C9, and CYP2E1) as well as UGT enzymes. For example, Kᵢ values were determined as ∼1.3 µM for CYP3A4 and ∼0.6 µM for UGT1A7 (Feng et al., 2022), indicating strong binding affinity. Although such plasma concentrations are unlikely to be achieved *in vivo*, these findings suggest a risk of drug–herb interactions when morusin or mulberry root bark extracts (rich in morusin) are co-administered with medications metabolized by the same enzymes.

In summary, UGT-mediated conjugation (via UGT1A and UGT2B isoforms) constitutes the primary elimination route for morusin, leading to excretable conjugates (except in cases of enterohepatic recycling). Phase I oxidation, mainly by CYP3A4, represents a secondary pathway responsible for generating hydroxylated metabolites (Liu et al., 2019). Supporting this, Liu et al. (2019) reported species-specific differences in morusin metabolism: eight Phase I metabolites were detected in animal liver microsomes, while six were identified in human liver microsomes. Despite these differences, CYP3A4 was confirmed as the main hepatic enzyme in humans, where CYP3A-mediated metabolism proceeds at a slower rate compared to animals, indicating an intrinsically lower clearance rate in the human liver.

### 7.5 Species differences in metabolism

Rather than being in kind, they are quantitatively different. This is evidenced by liver microsomes from rats, dogs, monkeys, minipigs, and humans: they all produce many of the same phase I metabolites (e.g., M1, M2, M5, M7) (Shi et al., 2016; Liu et al., 2019). Based on *in vitro* intrinsic clearance (CL_int) values reported by Shi et al. [16], the oxidative metabolism of morusin follows the order: minipigs > dogs > humans, indicating species-dependent differences in hepatic metabolic capacity (Shi et al., 2015). In line with this, the intrinsic clearance in human liver microsomes was but a fraction of that in rats or monkeys. Upon comparison, the major hydroxylated metabolite (“P1”) showed substantially less formation in human microsomes than in the animal microsomes (monkey and minipig 8–12 times higher, rats 6 times higher) (Feng et al., 2022). This suggests that humans can metabolize morusin very slowly by CYP pathways compared to some of the experimental animals. Human hepatic clearance is then predicted to be similarly lower, with morusin also likely to persist longer in humans than in rodents if it is not eliminated by UGT. Estimated hepatic clearance values of ∼8.3 mL/min/kg for humans contrast with ∼35.1 mL/min/kg for rats (Zhang X et al., 2021). Such differences emphasize the need for cautious scaling across species, from animal pharmacokinetics to humans. Conversely, glucuronidation, which, in some contexts, is considered the UGT-mediated transformation, appears to be less efficient in rats and, hence, also in humans. Such an event could then partially offset some differences towards total clearance. These species studies aim at giving an abundance of light: metabolically, as observed in humans, these species studies throw into consideration metabolic barriers while hinting at potential drug interactions that may be caused by morusin inhibition across CYP/UGT isoforms in various species (Panek-Krzyśko and Stompor-Gorący, 2021).

### 7.6 Excretion

With extensive conjugation, most of the morusin is excreted as metabolites. Glucuronide conjugates of morusin formed in the liver are readily excreted into the bile; thus, biliary excretion is a major route. Substantial amounts of the administered dose of morusin are recovered in the bile after rat dosing as M-4′-G and other glucuronides (Shi et al., 2015). These biliary metabolites may undergo enterohepatic recycling or be excreted in feces after reabsorption. Fecal excretion, as opposed to urinary, represents the predominant elimination route for morusin and its conjugates, particularly among flavonoids undergoing biliary clearance (Zhang F et al., 2021). A smaller fraction, at most, would appear as metabolites in the urine. While specific excretion data for morusin remains unsubstantiated, similarly, urine contains only a few glucuronides/sulfates during the elimination process. The 2018 rat study cited the presence of minute amounts of the oxidative metabolite of morusin in the plasma (Haywood and Glass, 2011), suggesting that renal excretion of unconjugated morusin might be minimal—and accordingly, any urinary excretion is expected to be of its water-soluble conjugates (Zhang F et al., 2021). Overall, the conversion of morusin to glucuronide moieties, followed by the excretion of those conjugates via bile (and feces), combined with enterohepatic recycling, prolongs the elimination tail. The plasma half-life (t_1_/_2_) of morusin in rats is up to a few hours; for instance, t_1_/_2_ was about 4–5 h for normal rats in one study (Shi et al., 2015).

### 7.7 Pharmacokinetics under pathological conditions

Indeed, the pharmacokinetics of morusin can be significantly altered by both physiological and pathological conditions. One very telling study compared its biosusceptibility within streptozotocin-induced diabetic rats versus normal rats. In streptozotocin-induced diabetic rats—the first well-characterized specimen of this chemically induced disease model—the LADME parameters indicated significantly higher systemic exposure to morusin than in healthy rats (Xiong et al., 2023). Once fed in a fixed dose as part of a flavonoid extract, threefold and twice higher mean values for AUC (325.0 ± 87.6 versus 116.4 ± 38.2 ng·h/mL) and Cmax were found in these animals, respectively, compared to their non-diabetic partners for sheer diabetic rats (Xiong et al.). These differences were pharmacokinetically significant, speaking. Likewise, other constituents of the extract also showed such differences, suggesting something more general: an association with glucose metabolism (Xiong et al., 2023). The reasons for what led to the change in PK under diabetes are not one, but several. The diabetic state may downregulate several metabolic enzymes and transporters, thereby prolonging drug survival in the organism. This is why morusin tended to exhibit a longer half-life (mean ∼9.8 h) in diabetic rats than in normal ones (∼4.3 h) (Xiong et al., 2023), although the ranges overlapped. It is possible that lower expression or activity of UGTs and CYPs in uncontrolled diabetes (Darakjian et al., 2021), together with gastroparesis (slower gastric emptying), results in altered absorption kinetics, leading to an elevated exposure (Dostalek et al., 2011). Additionally, liver and kidney dysfunctions contribute to reduced elimination (Xiong et al., 2023). All these data clearly show that pathological conditions (e.g., diabetes) would heighten the presence of morusin and possibly prolong its action, which may be a desirable quality for leading to efficacy or else pose a risk of accumulation according to the situation (Li et al., 2024). It is not surprising that no studies have yet evaluated the PK of morusin in other disease states (e.g., hepatic inflammation), but it is probably reasonable to assume that any condition affecting metabolic enzymes or excretory functions would also greatly affect morusin’s disposition.

It should be noted that these results underscore the importance of considering this molecule in different physiological states when developing therapeutics based on such findings. Possible dosing adjustments may be needed when applying these animal data to humans, especially in patients with metabolic disorders (Xiong et al., 2023).

## 8 Future directions

Despite the substantial body of preclinical research on morusin, its development as a therapeutic agent is still at an early stage. Most studies have been confined to *in vitro* experiments and animal models, leaving considerable uncertainty about its pharmacological behavior and clinical applicability in humans. To bridge this gap, future investigations must systematically address several critical areas, including human clinical validation, pharmacokinetic optimization, metabolite characterization, safety evaluation, and translational strategies.

### 8.1 Lack of human clinical data

The most critical limitation is the complete absence of human clinical studies. Current efficacy data are restricted to cell culture and animal models, making it impossible to draw firm conclusions about morusin’s safety, tolerability, and pharmacokinetic behavior in humans. Key questions remain unanswered, including its oral bioavailability, optimal dosing regimen, and potential toxicity profile. Future clinical studies should therefore include pharmacokinetic profiling in humans, ideally incorporating both intravenous (to determine absolute bioavailability) and oral administration. Furthermore, any signs of toxicity observed in preclinical studies—such as liver enzyme elevations—should be carefully evaluated during clinical development.

### 8.2 Pharmacokinetic optimization

Morusin’s poor aqueous solubility and extensive first-pass metabolism represent major barriers to its therapeutic use. Innovative formulation strategies could improve its bioavailability, such as nano-formulations, liposomal or niosomal carriers, prodrugs, or complexation with cyclodextrins, all of which may enhance systemic absorption (Haywood and Glass, 2011). For instance, Deng et al. (2017) developed morusin-loaded niosomes that improved drug delivery and enhanced anticancer activity. Future research should build on such approaches and explore medicinal chemistry modifications—for example, altering hydroxyl or prenyl moieties—to generate analogs with improved solubility and reduced metabolic clearance.

### 8.3 Activity of metabolites

The biological activity of morusin’s metabolites remains poorly defined. While the major glucuronide metabolite (M-4′-G) is presumed inactive—since glucuronidation generally deactivates xenobiotics—other oxidative metabolites may retain therapeutic activity. Detailed pharmacodynamic studies are needed to establish whether these metabolites possess anticancer or anti-inflammatory properties. If active metabolites are identified, they could themselves serve as novel drug candidates or inform combination approaches, such as co-administering glucuronidation inhibitors to increase metabolite exposure (though safety concerns must be rigorously addressed).

### 8.4 Potential for drug–drug interactions

Morusin has demonstrated potent *in vitro* inhibition of CYP3A4 and related metabolic enzymes, raising concerns about potential drug–drug interactions. While this effect may be minimal at low oral doses, it could become clinically significant with higher doses, intravenous administration, or advanced formulations that enhance systemic exposure. Consequently, *in vivo* interaction studies in animal models, followed by carefully designed human trials, are necessary to evaluate the risk of altered plasma levels of co-administered drugs.

### 8.5 Pharmacokinetics in special populations

Emerging evidence indicates that pathological conditions can alter morusin pharmacokinetics. For example, altered disposition in diabetic rats suggests disease states may modulate its metabolism and elimination (Xiong et al., 2023). Liver disease, renal impairment, age, sex, and gut microbiome composition may also influence its pharmacokinetics. In particular, gut microbial β-glucuronidase activity could significantly impact enterohepatic recycling by deconjugating glucuronides, thereby modifying systemic exposure. Future research should address these variables to ensure safe and effective use across diverse patient populations.

### 8.6 Towards clinical translation

For morusin to advance from preclinical promise to clinical utility, it must demonstrate the ability to achieve therapeutically relevant concentrations in target tissues while maintaining safety. One strategy could involve combining morusin with metabolic enzyme inhibitors or drug efflux transporter inhibitors to enhance tumor accumulation and antitumor efficacy (Deng et al., 2017). Although such approaches remain speculative, they exemplify the innovative pharmacological strategies required for clinical translation.

## 9 Conclusion

Morusin exhibits diverse pharmacological activities and unique structural features that position it as a promising lead compound for drug development. However, its clinical translation is hindered by poor solubility, low bioavailability, limited pharmacokinetic and safety data, and potential drug–drug interaction risks. To overcome these barriers, future research should prioritize advanced formulation strategies—such as nano- and lipid-based delivery systems to improve absorption and systemic exposure, alongside comprehensive toxicity assessments and early-phase clinical trials to establish dosing, safety, and efficacy in humans. Equally important are multidisciplinary efforts integrating pharmacology, medicinal chemistry, and clinical sciences to optimize pharmacokinetics, elucidate metabolite activity, and mitigate safety concerns. Strategic coordination of these approaches will be essential to accelerate morusin’s progression from preclinical promise to a clinically viable therapeutic option, ultimately realizing its potential as a novel agent derived from traditional medicine.
